# Ultrasonography and electrophysiological study of median nerve in patients with essential tremor

**DOI:** 10.1371/journal.pone.0215750

**Published:** 2019-04-23

**Authors:** Hye Lim Lee, Ji-sun Kim, Hanjun Kim, Il soo Kim, Jae-whan Kim, Ye-eun Kim, Seong-beom Koh

**Affiliations:** 1 Department of Neurology, Korea University Guro Hospital, Korea University College of Medicine, Seoul, Korea; 2 Department of Neurology, Mediplex Sejong Hospital, Incheon, Korea; Weill Cornell Medicine-Qatar, QATAR

## Abstract

Essential tremor (ET) is a common movement disorder characterized by postural or kinetic tremor. We aimed to evaluate median nerve enlargement in patients with ET using ultrasonography (USG). Thirty-eight hands from 19 patients with ET and 24 hands from 13 controls underwent nerve conduction studies (NCS) and USG at the wrist. Tremor severity was measured using the Fahn–Tolosa–Marin Tremor Rating Scale (FTM-TRS). The median nerve cross sectional area (mCSA) in USG and NCS parameters were compared using ANCOVA. We evaluated the correlation between mCSA and NCS parameters or FTM-TRS scores using linear regression analysis. mCSA was significantly larger (p<0.001) and NCS parameters were different in two groups. Also, mCSA was negatively correlated with part B and C scores of FTM-TRS (p<0.001 and p = 0.039, respectively). In conclusion, median nerve enlargement with the changes of NCS parameters was observed and correlated with the severity of tremor in patients with ET.

## Introduction

Essential tremor (ET) is a common movement disorder characterized by postural or kinetic tremor, and 85–95% of patients with ET exhibit involvement of the distal part of the upper extremities [1,2]. It usually presents as a bilateral postural 8–12 Hz tremor of the hands [3,4]. Overextended repetitive movement of the distal upper extremity could result in enlargement of the median nerve at the wrist because of cumulative trauma-related injury and these injuries may also lead to median neuropathy, including carpal tunnel syndrome (CTS) [5–7]. Thus, repetitive hand movement in ET could damage and lead to enlargement of the median nerve at the wrist.

High-resolution neuromuscular ultrasonography (USG) has been used to evaluate nerve disorders and is effective in evaluating superficial nerve conditions in a relatively low cost, quick, noninvasive, and dynamic assessment [8–10]. Morphologic parameters collected by USG, such as cross-sectional area (CSA) obtained from transverse scans, provide useful information complementary to electrophysiological data [11–13]. Nerve enlargement proximal to the site of entrapment is a common finding in entrapment neuropathies, and neuromuscular USG is suitable for confirming nerve enlargement [14–16].

We aimed to evaluate the enlargement of the median nerve at the wrist–specifically at the carpal tunnel inlet and outlet levels–in patients with ET in comparison with a healthy control group and the difference in nerve conduction study (NCS) parameters of median motor and sensory nerves between both groups. Moreover, we investigated the correlation between the enlargement of median nerve and electrophysiological parameters, clinical characteristics, and tremor severity in patients with ET.

## Methods

### Subjects

We included consecutive patients with ET and healthy volunteers prospectively from the Department of Neurology of the Korea University Guro Hospital from August 2015 to July 2016. The necessary sample size calculated using G-power 3.0, and we set the power as 0.95 considering a previous study in PD [8]. After the calculation, we needed 12 or more participants in each group; we included 19 patients in the ET group and 13 patients in the healthy control group. All patients in the ET group met the clinical diagnostic criteria for ET and included patients with bilateral symmetric postural or kinetic tremor of the hands in core symptoms in ET [2,17]. The healthy control group comprised age- and sex-matched participants who did not have any symptoms such as tremor, numbness, or paresthesia. In the ET group, patients who had diagnosed CTS before the onset of hand tremor were excluded. In both groups, subjects with any other abnormalities on NCS except entrapment neuropathy at the wrist were excluded. Neither ET patients nor healthy volunteers had any peripheral nerve disease including endocrine, metabolic, or toxic neuropathy. This protocol was approved by the regional institutional review board of Korea University Guro Hospital (IRB No. 2015GR0129).

### Clinical evaluation

We investigated the demographic and clinical information of participants including age, sex, height, weight, dominant hand, and disease duration. Participants underwent a neurologic motor and sensory examination in both hands, Phalen’s test, and Tinel’s test at the wrist. To assess the degree of tremor, we used the Fahn–Tolosa–Marin Tremor Rating Scale (FTM-TRS). FTM-TRS is the most widely used scale in ET [18]. The rating scale is divided into three parts (A, B, and C) and the subtotal score that yielded from each part can be summed up a total score or used separately in independent analyses. Part A (scores 1 to 9) mainly measured the severity of resting tremor with a steady posture on nine parts of the body. Part B (scores 10 to 14) particularly relates to action tremors of the hands or arms. Part C assesses functional disability. Its items evaluate the severity of tremor during daily life like speaking, eating, hygienic care, dressing, and working. The maximum possible scores are 80 for Part A, 36 for Part B, and 28 for Part C, making the maximum possible total score 144. In this scale higher scores reflect more severe symptoms [4].

### Electrophysiological study

In the NCS, median motor and sensory nerve conduction studies used the antidromic method, and were performed in the supine position on patients with ET and control volunteers using surface electrodes. The test was followed standard protocols of supramaximal stimulation and surface electrodes in both upper extremities. The technician was blinded to the clinical information of both groups. We assessed terminal latency and the amplitude of compound muscle action potential (CMAP) in median motor nerve, and the amplitude of sensory nerve action potential (SNAP) and nerve conduction velocity in median sensory nerve. The skin temperature of the participants was kept above 32°C.

### Ultrasonographic study

USG studies were performed by a board-certified physician with sufficient experience in neuromuscular sonography. The examinations were performed while blinded to clinical information and electrodiagnostic results. An Affiniti 70G ultrasound device (Philips Healthcare, Bothell, WA) with a 7–12 MHz linear array transducer was used for this study. The ultrasonographic gain was 45%, dynamic range was 62 dB, depth was 3.5 cm, mechanical index was 1.3, and thermal index (for soft tissue) was 0.2. Each participant was lying in the supine position and the elbow and wrist were fully extended. The transducer of USG was first used to check a transverse view of the distal wrist crease. The CSA of the median nerve (mCSA) was calculated with a direct method using a continuous boundary trace just within the echogenic boundary of the nerve at the carpal tunnel and mid-forearm levels [19]. We were careful enough not to give additional pressure from the probe on the nerve. Median nerve enlargement was confirmed according to the American Association of Electrodiagnostic Medicine criteria [20].

### Statistical analysis

An analysis of covariance (ANCOVA) was used to compare mCSA and NCS parameters to adjust age and heights, and the chi-square test was used to compare demographic features and the number of participants with median nerve enlargement between the ET and healthy control groups. In patients with ET, we assessed the correlation between mCSA at the wrist level and other variables, such as NCS parameters or FTM-TRS scores, using a multiple linear regression analysis with a forward step method. The data analyses were performed using SPSS version 22.0 (SPSS Inc., Chicago, IL, USA). The data are presented as the mean ± standard deviation (SD) values, and p < 0.05 was considered indicative of statistical significance.

### Ethical publication statement

The authors confirm that they have read the Journal’s position on issues involved in ethical publication and affirm that this report is consistent with those guidelines.

## Results

### Demographics

A total of 38 hands from 19 patients with ET (four male and 15 female) and 24 hands from 13 healthy controls (six male and 7 female) were included in this study. The mean age of the two groups showed statistical difference (57.95±15.35 years in patients with ET and 45.54±13.13 years in healthy controls, p = 0.024) and the mean height showed difference (158.21±9.10 centimeters in patients with ET and 165±8.90 centimeters in healthy controls, p = 0.045). The median disease duration of the patients with ET was 72 months (range, 3–360 months) and mean FTM-TRS score was 33.26±20.71 (Table 1 and S1 Dataset). On neurologic examination, paresthesia of both hands was found in four patients, and one had positive results for both Tinel’s test and Phalen’s test over both median nerves. No patient exhibited any hand weakness.

**Table 1:** Clinical characteristics and NCS parameters of patients and controls.

	Patients (n = 19)	Controls (n = 13)	p-value
Sex (male, n [%])	4 [21.1%]	6 [46.2%]	0.244
Age (mean±SD)	57.95±15.35	45.54±13.13	**0.024**
Height (cm, mean±SD)	158.21±9.10	165.00±8.90	**0.045**
Weight (kg, mean±SD)	61.58±10.49	66.0±1.41	0.567
Body mass index (kg/m^2^, mean±SD)	24.61±3.83	24.12±3.18	0.866
Disease duration (month, median [min, max])	72 [3, 360]		
NCS^a^ parameters			
Median motor terminal latency (ms, mean±SD)	3.22±0.50	2.87±0.33	**0.018**
Median motor amplitude (μV, mean±SD)	10.3±2.56	11.8±2.87	**<0.001**
Median sensory amplitude (μV, mean±SD)	47.37±32.26	69.46±32.11	**<0.001**
Median sensory conduction velocity (m/s, mean±SD)	44.39±5.46	49.46±3.24	**<0.001**
FTM-TRS^b^ (mean±SD)			
Part A	13.16±7.99		
Part B	12.42±8.24		
Part C	7.68±5.69		
Total	33.26±20.71		

^a^NCS nerve conduction study

^b^FTM-TRS Fahn–Tolosa–Marin Tremor Rating Scale

### Electrophysiological findings

The motor terminal latency, motor CMAP amplitude, median sensory ampltidue, and sensory conduction velocity of median nerve in patients with ET and healthy controls were significantly different based on ANCOVA with adjustment of age and height. Median motor terminal latency was longer (p = 0.018), motor and sensory amplitude were smaller (p<0.001), and sensory amplitudemedian sensory nerve velocity was slower (p<0.001) in patients with ET than in the healthy control group (Table 1).

### Comparison of mCSA at the carpal tunnel level between patients with ET and controls

**Fig 1:**
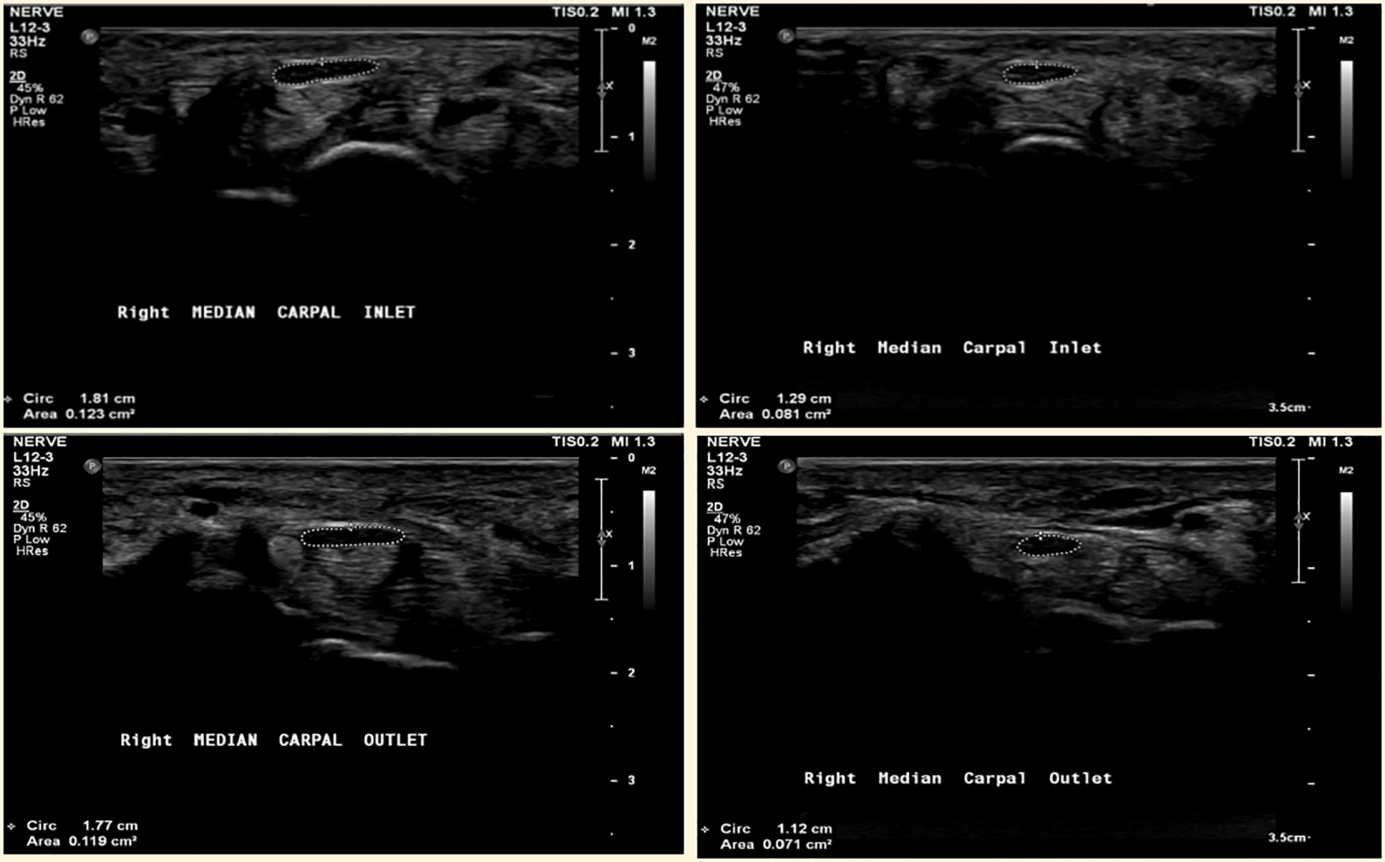
Ultrasound image of the median nerve cross-sectional area (CSA) at the carpal tunnel level. Median CSA of patient (A, B) and healthy control (C, D).

USG at the carpal inlet level found that the mCSA of patients with ET (9.51±2.43 mm^2^) was significantly larger than that of controls (7.71±1.03 mm^2^, p = 0.001; Table 2 and Fig 1). At the level of the carpal outlet, a difference was also seen between both groups (9.15±1.75 mm^2^ and 7.46±0.99 mm^2^, p<0.001; Table 2). The ratio of the carpal inlet and outlet to mid-forearm in the patients with ET was showed higher than that in the healthy control group (p<0.001 and p = 0.001, respectively).

**Table 2:** Comparison of mCSA at the wrist level between patients with ET and controls.

	ET (n = 38)	Control (n = 24)	p-value
Carpal inlet	9.51±2.43	7.71±1.03	**0.001**
Carpal outlet	9.15±1.75	7.46±0.99	**<0.001**
Carpal inlet / mid-forearm	1.69±0.39	1.36±0.19	**0.001**
Carpal outlet / mid-forearm	1.63±0.28	1.31±0.18	**<0.001**

### Correlation between the mCSA and electrophysiological findings

The mCSA at both carpal inlet and outlet levels was correlated with median motor terminal latency, median sensory nerve conduction velocity and amplitude based on multiple linear regression analysis with a forward step method. The mCSAs were negatively correlated with the sensory conduction velocity of the median nerve, and were positively correlated with median motor terminal latency at the carpal inlet and outlet levels (Table 3). The median CMAP amplitude was negatively correlated with mCSA at carpal outlet levels. The median sensory amplitude was showed negative correlation with mCSA at carpal inlet level, however, positive correlation with mCSA at carpal outlet level. Age was negatively correlated with mCSA at carpal inlet level (Beta coefficient -0.115 [95% CI, -0.155 to -0.075, p<0.001] and outlet level (Beta coefficient -0.053 [95% CI, -0.085 to -0.020, p = 0.002]). Each variable was analyzed with adjustments for age and height.

**Table 3:** Correlation between mCSA at the wrist level and other variables in patients with ET.

Variables	Carpal inlet		Carpal outlet	
	Unstandardized coefficient	p-value	Unstandardized coefficient	p-value
Tremor severity				
TRS, part A^a^	-	-	-	-
TRS, part B^a^	-0.163	**< 0.001**	-	-
TRS, part C^a^	-0.140	**0.039**	-	-
TRS, total*	-	**-**	-	-
NCS, median				
CMAP, TL	2.838	**<0.001**	1.889	**< 0.001**
CMAP, amplitude*	-	-	-0.027	**0.010**
Sensory, NCV	-0.269	**< 0.001**	-0.170	**0.001**
Sensory, amplitude	-0.044	**< 0.001**	0.198	**0.046**

For multiple linear regression analysis, each variable was respectively analyzed with the following variables; age, height, body mass index, disease duration. Among the variables, age was significantly correlated with median CSA.

a:Non-significant variables were excluded from final regression model, hence the unstandardized coefficient and p-value could not be demonstrated.

### Correlation between the tremor severity and ultrasonographic findings

The mCSA at the carpal inlet level was negatively correlated with the part B and part C FTM-TRS scores (p<0.001 and p = 0.039, Table 3). However, the mCSA at the carpal outlet level and FTM-TRS scores were not significantly correlated. FTM-TRS and total scores were not correlated with mCSA at the carpal inlet and outlet levels.

## Discussion

We suggest that abnormal repetitive movement of the hands may result in nerve injury and subsequent CSA increase in patient with ET. We performed a study comparing the mCSA at the wrist between patients with ET and healthy controls. The mCSA in patients with ET was larger than that in healthy controls based on static and NCS parameters; motor terminal latency, CMAP amplitude, sensory SNAP amptlidue, and sensory velocity in the median nerve were significantly different between the patients with ET and healthy controls. We also evaluated the association between mCSA and tremor severity with FTM-TRS. The mCSA at the carpal inlet level was negatively correlated with part B and part C FTM-TRS scores in patients with ET.

In previous studies, tremor was associated with CTS with median nerve enlargement identified using USG and the degree of tremor was correlated with mCSA in Parkinson’s disease (PD). However, there are no investigations of the relationship between tremor and ultrasonographic and electrophysiological findings in ET. In this study, we confirmed that there was median nerve enlargement at the carpal tunnel inlet and outlet levels in patients with ET using neuromuscular USG. We adjusted age and height because of statistical differences in two groups. The median nerve enlargement (mCSA ≥9 mm^2^) was found in nine patients with ET and two participants in the control group (p = 0.004) [21]. The mechanism for median nerve enlargement in ET was unclear; however, overextended repetitive movement of the distal upper extremity might cause enlargement of the median nerve at the wrist through cumulative trauma-related injury and this injury may also lead to median nerve enlargement even without a “pill-rolling tremor” like that seen in PD [5–8]. The wrist-forearm ratio is very sensitive in detecting median nerve enlargement at the carpal tunnel levels, and when it is 1.4 or higher, it usually indicates CTS. The sensitivity of the wrist to forearm ratio in patients with CTS was reported to be 100%, while using only mCSA at the wrist results in a sensitivity of 45–93% depending on the cutoff value used [22]. In our study, the average carpal inlet to mid-forearm and carpal outlet to mid-forearm ratio were increased, 1.69 and 1.63, respectively, and the ratio was within the normal range in the control group (1.31).

We also compared median motor terminal latency, motor and sensory amplitude, and sensory conduction velocity between the two groups after adjustment of age and height. In patients with ET, only four patients showed prolonged terminal latency with slow sensory velocity in the median nerve, and one patient showed slow median sensory nerve conduction velocity in NCS with respect to our reference values. However, the average median sensory nerve velocity was slower, and median motor terminal latency was longer than in the healthy control group, although most participants were within the normal range of reference values.

The median nerve enlargement at the wrist detected using neuromuscular USG was also correlated with electrophysiological parameters. In the multivariable regression analysis, mCSA at the carpal inlet and outlet levels were shown to be negatively correlated with sensory conduction velocity of the median nerve, and mCSA at the carpal outlet and inlet levels were positively correlated with the median motor terminal latency. These findings match the electrophysiologic findings in CTS, which is a typical disease of median nerve injury at the wrist level; sensory nerve conduction velocity is especially known as a sensitive marker to diagnose the early stages of CTS [23]. However, sensory amplitudes of the median nerve vary among individuals and have limited diagnostic value in CTS until the amplitude decreases to under the normal range of reference value [24]. Thus, the relationship between mCSA at the carpal tunnel level and sensory amplitude needs further evaluation in a large sample size study.

Nerve conduction velocity and amplitudes of CMAP or SNAP in the median nerve are known to reflect median nerve damage; thus, it might be related to median nerve dysfunction caused by repetitive movements in the patient with ET [25]. We evaluated the severity of tremor FTM-TRS, which consists of three parts: part A, part B, and part C. Among these parts, part B evaluated the action tremor in both arms and part C was related to functional disability in activities of daily living. The higher the score for part B and C, the more severe the tremor and disruption of activities of daily living in the patient with ET. Unlike part A that evaluates resting tremor, part B and C may reflect the use of the affected hand especially in ET patients. Thus, the mCSA with severe tremor might tend to be smaller than that without severe tremor by restraining the use of the hand by itself.

A recent case was presented that dystonic tremor improved after operation for carpal tunnel release [26]. However, the case was induced the hand tremor in a specific posture with flexion at wrist, unlike postural tremor in the patient with ET. Also, we excluded the patients who had the symptoms of CTS before the onset of hand tremor in our study. Further evaluations were needed to determine the relationship with symptomatic CTS, rather than simply median nerve enlargement.

This study has several limitations. We have a small sample size, and there was not complete blinding because patients’ tremors were easily visible. In addition, we did not investigate the occupational history or habitual movement that might be related to median nerve enlargement. However, we investigated median nerve enlargement and its association with the degree of tremor in ET for the first time. Further evaluation of the progression to symptomatic CTS from asymptomatic median nerve enlargement is required in future in the large prospective study.

In conclusion, we demonstrated that mCSA at the wrist in patients with ET was significantly larger than that in a healthy control group as confirmed using neuromuscular USG, and that NCS parameters were also different between the two groups. Tremor severity was negatively correlated with mCSA, although the mechanism was still unclear. ET could be related to secondary peripheral nerve damage and appropriate assessment and management of such secondary problems should not be neglected.

## Supporting information

S1 Dataset The minimal data set for ET patients. (SAV)

## Abbreviations

ET: Essential tremor
CTS: carpal tunnel syndrome
NCS: nerve conduction studies
USG: ultrasonography
CSA: cross-sectional area
mCSA: median nerve cross-sectional areas
FTM-TRS: Fahn–Tolosa–Marin Tremor Rating Scale
